# Endoplasmic Reticulum Stress and Allergic Diseases

**DOI:** 10.1007/s11882-017-0751-9

**Published:** 2017-11-08

**Authors:** Jae Seok Jeong, So Ri Kim, Seong Ho Cho, Yong Chul Lee

**Affiliations:** 1 Department of Internal Medicine, Research Center for Pulmonary Disorders, Chonbuk National University Medical School, san 2-20, Geumam-dong, Deokjin-gu, Jeonju, 561-180 South Korea; 2 Research Institute of Clinical Medicine of Chonbuk National University-Biomedical Research Institute of Chonbuk National University Hospital, San 2-20 Geumam-dong, Deokjin-gu, Jeonju, 561-180 South Korea; 3 Division of Allergy and Immunology, Internal Medicine, Morsani College of Medicine, University of South Florida, Tampa, USA

**Keywords:** Precision medicine, Biomarker, Allergic diseases, Endoplasmic reticulum stress, ER stress, Inflammation

## Abstract

**Purpose of Review:**

In this review, we will integrate recent knowledge on endoplasmic reticulum (ER) stress and allergy, thereby highlighting the therapeutic potential of ER stress in the context of precision medicine for allergic diseases.

**Recent Findings:**

Emerging evidence suggests that allergic diseases are very heterogeneous having numerous endotypes. This leads to the new era of modern medicine, which assumes that a particular endotype-driven therapy, called precision medicine, would be more efficacious in a specific group of patients rather than in all patients. Currently, a dichotomy involving type 2/non-type 2 immune response underlies most of the studies on inflammatory and immunologic mechanisms of allergic disorders. Whereas there are several approved or investigational endotype-driven therapeutic agents targeting type 2 immune responses, investigation of mechanisms and endotype-driven interventions regarding non-type 2 immune response lags far behind.

**Summary:**

Considering that non-type 2 immune response may represent a significant proportion of allergic disease, particularly corticosteroid-resistant severe disease, defining a novel concept of endotype-driven approach may be essential. Recently, stress responses originate from the endoplasmic reticulum (ER) and the associated inflammatory molecular platform has been suggested as a crucial player of immune and inflammatory responses. This implies that ER stress-related pathways may represent a new endotype-driven therapeutic strategy in the treatment of allergic diseases.

## Introduction

It is now believed that allergic diseases including bronchial asthma are very heterogeneous having numerous endotypes according to unique genetic, pharmacologic, physiologic, biologic, and immunologic mechanisms. In this context, optimal symptom control and successful treatment of allergic diseases may necessitate tailored approaches based on distinct pathophysiology in selected patients [1, 2•]. Thus, increasing awareness of heterogeneity in allergic diseases has coincided with the emergence of endotype-driven approaches, called precision medicine [3, 4•]. This new era of medical treatment highlights that specific group of patients may have a better response to some drugs, whereas no single drug would be efficacious for all patients. In addition, validated and qualified biomarkers are essential to identify a certain endotype better, so that researchers can define populations that will derive the most benefit from a drug [5]. For instance, early belief that bronchial asthma is the hallmark of type 2 helper T (T_H_2) cell-mediated process led to clinical trials on evaluating therapeutic effects of anti-interleukin (IL)-5 on broad spectrum of severe asthmatics [6, 7]. Initially, it failed to show significant benefits on the clinical course of the disease because inclusion of study participants was carried out in an unselected manner involving all types of asthmatics. However, later analysis revealed that anti-IL-5 can offer substantial clinical benefits in a subset of severe asthma patients with significant blood eosinophilia [8, 9]. Therefore, to define the endotype with a stable pattern of biologic mechanism in allergic disorders will be critical for selecting any therapeutic modality more efficiently.

The endoplasmic reticulum (ER) is the largest intracellular organelle and is crucial for cellular Ca^2+^ storage/homeostasis and the assembly, folding, and transport of soluble/membrane proteins. Particularly as a key regulator of protein homeostasis essential for cell survival and function, ER is abundantly equipped with chaperones and enzymes that facilitate proper folding of the client proteins. Meanwhile, maintaining optimal internal conditions of ER is essential for proper folding of proteins, because ER chaperones and enzymes are highly sensitive to various stresses such as perturbations in redox state, Ca^2+^ concentration, and cellular energy levels [10]. Thus, altered ER homeostasis may lead to imbalance between the ER protein folding capacity and the folding load of nascent proteins, and the accumulation of misfolded/unfolded proteins in the ER lumen occurs, which is referred as ER stress. Misfolded/unfolded proteins are toxic to cells and can cause cellular stress and cell death. To maintain cellular homeostasis against this crisis, cells have evolved to efficiently detect ER stress through ER transmembrane sensors, all of which eventually trigger adaptive unfolded protein response (UPR) to restore ER functionality. Notably, a vast amount of recent study has also revealed that ER stress and UPR intersect with immunity and inflammation [11, 12], thereby making them as valuable therapeutic targets for treating many human disorders [13]. Particularly, emerging evidence also suggests that ER stress and UPR is closely associated with allergic inflammation through engaging with many cellular inflammatory platforms [14••].

In this review, we will summarize the current concept of the pathogenesis of allergic diseases focusing on their heterogeneity in underlying immunological basis. Besides, we will highlight the therapeutic potential of ER stress and UPR in the context of precision medicine for allergic diseases through integrating the recent advances in our knowledge on this field of research.

## Heterogeneity in Allergic Diseases (Type 2 and Non-type 2)

Better management of allergic diseases can be achieved through recognizing the disease heterogeneity, which is composed of diverse pathogenic mechanisms leading to clinically significant outcomes. A myriad of pathways implicated in allergic diseases have been reported to date. However, most of them may fall into type 2 or non-type 2 response based on underlying inflammatory and immunologic mechanisms. Further, several sub-endotypes may exist within each of them according to dominant cell types that orchestrate immune responses [4•].

### Type 2 Immune Response

Type 2 allergic immune responses are typically characterized by eosinophilic inflammation and associated with increases of type 2 cytokines including interleukin (IL)-5, IL-13, and IL-4 in blood and affected tissues. Generally, type 2 immune responses are closely associated with atopy/allergy, in which the presence of serum antigen-specific immunoglobulin E (IgE) (mainly driven by IL-4) is the hallmark of adaptive immunity involving type 2 helper T cells (T_H_2 cells). This immune pathway has been reported to be corticosteroid (CS)-sensitive and essential in many allergic diseases including allergic asthma, allergic rhinitis, and atopic dermatitis [4•]. However, type 2 responses may also be generated by other mechanisms irrespective of IgE reactivity to allergens and T_H_2 cells, as clearly demonstrated in recent researches on bronchial asthma and allergic rhinitis. In the non-allergic mechanism of type 2 cytokine production, particularly in bronchial asthma, chronic airway epithelial activation in relation to environmental factors (e.g., pollutants, irritants), viral infections, or fungal exposure induces epithelial production of IL-25, IL-33, and thymic stromal lymphopoietin (TSLP). Innate lymphoid cells (ILCs) react to these epithelium-derived cytokines, thereby producing IL-5 and IL-13 associated with airway eosinophilia and bronchial hyper-responsiveness, respectively. This mechanism may represent asthma patients who have non-atopic/allergic and severe CS-insensitive disease while possessing eosinophilic type 2 immune responses in tissues [15]. Furthermore, the presence of this sub-endotype of type 2 immune responses partly explains why targeting type 2 cytokine pathway is effective in a subset of non-atopic asthmatic patients with high levels of eosinophils in blood and frequent exacerbation despite maximal current treatments including inhaled CS and/or systemic CS [16, 17]. Based on these findings, currently, there are several approved or investigational endotype-driven therapeutic agents targeting type 2 immune responses in bronchial asthma and other allergic diseases [18, 19]. In addition, diverse innate and adaptive immune pathways related to T_H_1/T_H_17 cells, environmental exposure (e.g. smoking, occupational exposure), viruses/bacteria, and tissue injury may further modulate type 2 immune responses, particularly in less allergic form, leading to clinically more severe allergic disease with mixed eosinophilic/neutrophilic inflammation in some individuals [20, 21•, 22, 23]. These findings also highlight the complex nature of the CS-resistant severe allergic inflammatory process.

### Non-type 2 Immune Response

Currently, the overall proportion of asthma associated with type 2 immune responses is estimated to be approximately 50% of patients [24, 25]. Thus, individuals with non-type 2 immune responses represent a large proportion of asthmatics. Likewise, results from several previous clinical trials demonstrating the ineffectiveness of type 2 cytokine-targeted therapies in non-phenotyped, overall groups of asthmatics may imply the presence of bronchial asthma having non-type 2 immune response [26]. However, little is understood regarding the mechanisms underlying this type of immune response and most of the knowledge is derived from studies of bronchial asthma and rhinitis. Generally, both type 17 (T_H_17) and type 1 (T_H_1) responses are often associated with neutrophilic inflammation of airways. While lung neutrophilia can be interpreted as secondary finding of CS use [27], the presence of neutrophilic inflammation has been associated with more severe clinical manifestations of bronchial asthma, more CS-refractory disease, and lower lung function in affected patients [21•, 28•]. In a preclinical model of asthma, adoptive transfer of allergen-specific T_H_17 cells to mice induced a chemokine (CXCL8, also known as IL-8)-mediated neutrophil influx into the lung, which was not attenuated by CS [29]. Similarly, sputum *IL17A* and *IL8* mRNA levels were correlated with each other and with sputum neutrophil counts in asthmatics and levels of these transcripts increased with increasing severity of asthma [30]. However, single targeted therapy blocking IL-17 receptor signaling has shown a minimal effect in subjects with inadequately controlled moderate to severe asthma in a clinical trial [31]. These results may be in line with the hypothesis that CS-insensitive severe asthma possesses mixed type 17/type 1 immune response in the background of variable type 2 immunity [21•, 32]. In the same vein, the existence of a unique molecular phenotype of asthma characterized by simultaneous activation of type 17 and type 1 immune response with airway neutrophilia has been demonstrated in clustering analysis of sputum cell transcriptomics from moderate to severe asthmatic subjects [33•]. In fact, early reports showed that interferon (IFN)-γ producing T cells were increased in airways of asthmatics [34] and serum concentration of IFN-γ was elevated especially in patients with acute severe asthma [35]. More recently, IFN-γ has been implicated in bronchial asthma pathogenesis through T_H_2-independent IFN-γ/mast cell axis [36] as well as its classical effects on T_H_2 cells [37, 38]. However, little is known about the therapeutic effect of IFN-γ blockade in the treatment of bronchial asthma and other allergic diseases so far. Furthermore, considering the existence of another largely unknown non-type 2 paucigranulocytic asthma (the absence of detectable inflammatory process) [22, 33•], development of effective endotype-driven therapy may be further hampered by our limited knowledge on the mechanisms contributing to the non-type 2 immune response in allergic diseases. Currently, there is no approved endotype-driven therapeutic agent, targeting non-type 2 allergy [18].

## ER Stress and the UPR Pathways

Three ER transmembrane sensors, including inositol-requiring enzyme 1α (IRE1α), double-stranded RNA-dependent protein kinase (PKR)-like ER kinase (PERK), and activating transcription factor 6 (ATF6), monitor protein homeostasis of ER lumen and transmit their information to the cytosolic compartment of cells through UPR pathways. This process can be both normal physiology and pathological phenomenon because even in normal physiological processes, such as increasing demands of protein secretion in secretory cells (e.g., plasma cells producing a large amount of immunoglobulins), cells can experience ER stress. Therefore, the canonical understanding is that UPR fine-tunes the secretory pathway of ER and attempts to reduce ER stress through reducing demand of protein folding, promoting ER-associated degradation of proteins by the ubiquitin-proteasome system (namely ER-associated degradation, ERAD), and increasing ER chaperones and enzymes helping protein folding to defend cells from ER stress. If cells fail to resolve ER stress, these adaptive responses will initiate apoptosis. Recently, in addition to these canonical UPR activities, non-canonical UPR activities are involved in connecting protein homeostasis-related cellular apparatus to a wide array of cellular events including immunity and inflammation through various mechanisms, as substantially reviewed elsewhere [11, 12].

**Fig. 1:**
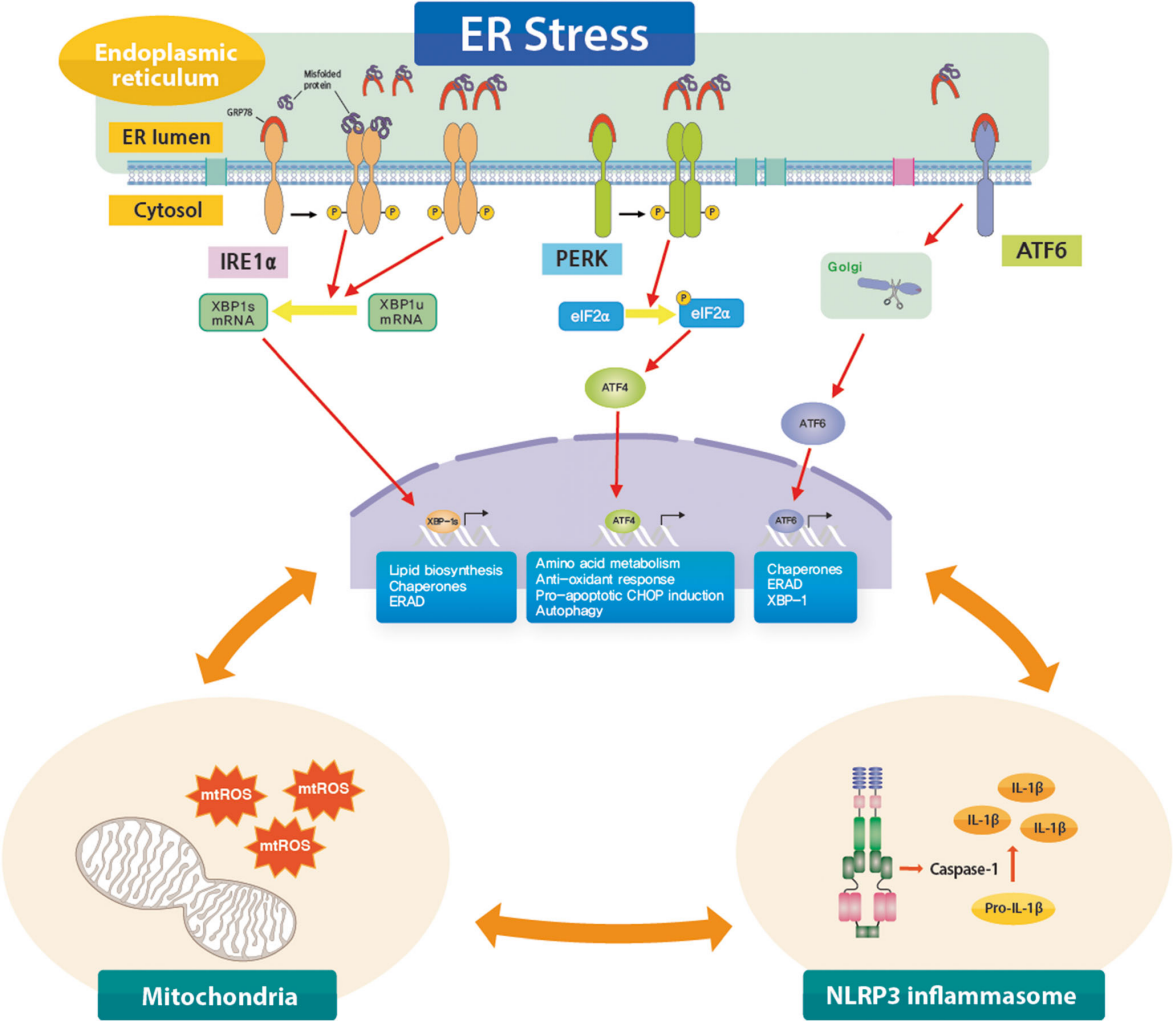
Interconnection between endoplasmic reticulum (ER) stress/unfolded protein response (UPR) pathways and mitochondria-NLRP3 inflammasome in allergic diseases. The accumulation of misfolded/unfolded proteins in the ER lumen activates UPR, which is mediated by three ER transmembrane stress sensors including inositol-requiring 1α (IRE1α), double-stranded RNA-dependent protein kinase (PKR)-like ER kinase (PERK), and activating transcription factor 6 (ATF6). In a condition of ER stress, an abundant ER chaperone, glucose-regulated protein 78 (GRP78), preferentially associates with accumulated misfolded/unfolded proteins. Dissociation of GRP78 from the ER stress sensors, or direct engagement of misfolded/unfolded protein to IRE1α, transmits signals about folding status of ER to the cytosol and nucleus. The canonical aspect of UPR regulates the secretory pathway of ER and attempts to reduce ER stress through reducing demand of protein folding, promoting ER-associated degradation (ERAD) and gene expression involved in cell survival (e.g., autophagy), and increasing ER chaperones to defend cells from ER stress. If cells fail to resolve ER stress, these adaptive responses will initiate apoptosis, mainly through C/EBP homologous protein (CHOP). In addition to this canonical aspect of UPR pathways, close interrelationship between ER/UPR pathways and cellular inflammatory platforms including mitochondria (e.g., oxidative stress from mitochondrial reactive oxygen species (ROS); mtROS) and NLRP3 inflammasome (an interleukin-1β producing platform) may be critically implicated in a unique form of corticosteroid-resistant type 2 allergic immune response

IRE1α is the most evolutionarily conserved sensor pathway among three UPR pathways and possesses both protein kinase activity and site-specific endoribonuclease (RNase) activity. In the presence of ER stress, IRE1α is activated when an abundant ER chaperone glucose-regulated protein 78 (GRP78) dissociates from IRE1α. Similar mechanisms (ER stress-driven dissociation of GRP78) also explain the activation of PERK and ATF6. Direct activation of IRE1α following engagement with misfolded proteins has been also demonstrated. Dissociated GRP78 preferentially binds to unfolded/misfolded proteins allowing IRE1α to dimerize and autophosphorylate through its kinase activity. This leads to the activation of specific RNase activity of IRE1α, leading to the splicing of mRNA encoding X-box-binding protein 1 (XBP1u) and generating a spliced variant (XBP1s). XBP1s functions as a transcription factor for genes associated with lipid metabolism, immune and inflammatory responses, and cellular differentiation as well as genes traditionally associated with structural and functional expansion of ER and ERAD [39] (Fig. 1). Furthermore, through its non-specific RNase activity, IRE1α has known to degrade ER-membrane-associated mRNA to reduce protein production, also known as regulated IRE1α-dependent decay [40].

Activation of PERK, regulated similarly to IRE1α, leads to the recruitment and phosphorylation of a translation initiation factor, eukaryotic translation initiation factor 2α (eIF2α), through its kinase activity. Phosphorylation of eIF2α inhibits synthesis of proteins through interfering the assembly of the ribosome in eukaryotic cells, thereby reducing protein folding load in ER-stressed cells. In addition, phosphorylated eIF2α selectively induces translation of ATF4 mRNA that contains regulatory sequences such as an inhibitory upstream open reading frame. As a transcription factor, ATF4 controls amino acid metabolism, anti-oxidant response, and autophagy (an adaptive self-eating process by which cellular components are encapsulated in autophagosomes and degraded). Furthermore, PERK-mediated apoptosis occurs later during ER stress when causative stimuli of ER stress is strong and the other arms of UPR fail to restore protein homeostasis. This process is known to be mediated by C/EBP homologous protein (CHOP), which is one of the representative ER stress markers in addition to GRP78 [41•] (Fig. 1).

Lastly, on release from GRP87, ATF6 moves to the Golgi apparatus where it is cleaved to produce the functional cytosolic fragment of ATF6. Then, fragmented ATF6 is translocated to the nucleus and activates expression of chaperones and ERAD-associated proteins (Fig. 1). Many aspects of outcomes from ATF6 activation resemble those of IRE1α/XBP1 pathway on the ER protein quality control system. Moreover, complete activation of the IRE1α pathway is partly controlled by ATF6 pathway because further induction of XBP1 mRNA depends on ATF6 [42]. Thus, the ATF6 pathway seems to fine-tune UPR along with IRE1α and PERK pathways.

## ER Stress as an Endotype of Severe Allergic Inflammation

### Involvement of ER Stress and UPR in Various Facets of Allergic Response

The allergic response begins in the interface between external environment and epithelium and subsequently involves diverse cell types at all levels of innate and adaptive immunity. During these processes, cells produce large amounts of secretory proteins to defend themselves against endogenous and exogenous threats and/or to efficiently communicate with other cell types for generating an organized immune response. Thus, proper functioning of ER and maintenance of protein homeostasis is critical in these cells. Although the presence of ER stress is not always interpreted as a pathological phenomenon [43], sustained and overwhelming ER stress in various cells can profoundly impact normal cellular physiology. Importantly, the influence of ER stress is just restricted to protein folding, but can intersect at many levels with immunity, thereby leading to complex chronic inflammatory disease such as allergy.

#### Epithelial Cell-Dendritic Cell (DC) Interaction

Before activation of various T cells implicated in various endotypes in allergic diseases, antigen-presenting DCs must recognize allergen and present it to T cells in draining lymph nodes. Epithelial cells are known to be a key modulator in controlling DC activation through releasing epithelial-derived cytokines (e.g., IL-25, IL-33, TSLP) and endogenous-associated molecules (e.g., uric acid, ATP, HMGB1). Given the role of epithelial cells as the first line of defense, coexistence of diverse environmental insults (e.g., pollution, smoking, occupational exposure, viruses/bacteria, and simultaneous exposure to other allergens) may also converge on epithelial-DC interaction [15, 44], further shaping and characterizing the underlying endotype of allergic response. In this regard, the role of protein-secreting apparatus of epithelium is indispensable. For example, inflamed airway epithelial cells demonstrate overt signs of ER stress [45] and bronchial epithelial XBP-1 has been reported to mediate inflammation-induced ER/Ca^2+^ store expansion which amplifies Ca^2+^-dependent secretion of cytokines [46]. Moreover, the IRE-1/XBP-1 arm of UPR has been proposed to be important in maintaining the integrity of airway [47] and intestinal epithelium [48]. Very recently, airway epithelial ORM (yeast)-like protein isoform 3 (ORMDL3), an ER transmembrane protein associated with *ORMLD3* gene locus (17q21) well-known as a highly significant risk factor for the development of asthma [49, 50], has been demonstrated to be critically implicated in fungal allergic airway disease through ATF6-mediated activation of XBP-1 and ERAD pathway [51••]. Meanwhile, IRE1α-XBP1 is also a prerequisite for the proper antigen-presenting function of DC as well as DC development and survival in physiologic context [11, 52, 53]. Taken together, ER stress and UPR pathway may be a central player in the regulation of epithelial-DC interaction that is crucial for the initiation and amplification of allergic response.

#### B Cells, Plasma Cells, and T Cells

Activation of adaptive immunity involving various T cells and B cells (including secretory plasma cells which secrete high levels of antibody), which contribute to diverse endotypes of allergic response, follows the initial recognition and amplification phase that involves epithelial-DC interaction. In the draining lymph nodes, T_H_2 and T_H_17 polarization may occur from naïve T cells with the help of migratory CD11b^+^ conventional DCs. Subsequently IL-4 production from T_H_2 cells induces class switching in B cells, and synthesis of IgE from plasma cells which contributes to allergen-specific sensitization for type 2 immune response. Once sensitized, repeated exposure to the same offending allergen may lead to the robust re-stimulation of these effector cells that is mainly mediated by poorly migratory CD11c^hi^ monocytic DCs [15]. In this process, XBP1 is known to be pivotal in terminal differentiation of B cells into highly secretory plasma cells through mediating expansion of the ER and synthesis of proteins required for antibody production and secretion [54]. Moreover, the IRE1α-XBP1 arm of UPR is thought to be important in the early developmental stage of B cells [55] and terminal differentiation of effector CD8^+^ T cells [56]. Notably, considering that XBP-1 mRNA in naïve B cells is uniquely induced by IL-4 [57], this arm of UPR may be particularly important in type 2 endotype of allergic response.

#### Granulocytes and Macrophages

Eosinophils and neutrophils are major effector cells in type 2 or non-type 2 immune response, respectively. However, neutrophilic inflammation may coexist with variable degrees of eosinophilic inflammation, particularly in a more severe form of the allergic disease with mixed eosinophilic/neutrophilic inflammation [20]. Alternatively activated macrophages may further fine-tune these processes through upregulation of chitinase-like proteins, one of the most abundant proteins under type 2 immune response that are involved in impaired anti-viral immunity [58] and neutrophil-rich type of allergic inflammation under type 2 immunity [59]. Among diverse granulocytes, differentiation of eosinophils from progenitors of myeloid cells is known to uniquely rely on the IRE1α-XBP1 pathway and deletion of XBP1 results in massive defects in eosinophil maturation [60••]. Additionally, macrophages are known to associate cell surface innate toll-like receptor (TLR) signaling with intracellular IRE1α-XBP1 pathway-mediated secretion of pro-inflammatory cytokines (e.g., IL-6 and tumor necrosis factor) [61], thereby linking ER and UPR pathway to innate effector function in macrophages. Furthermore, at the same time, TLR signaling suppresses the ATF4-CHOP-mediated cellular apoptotic pathway to effectively coordinate innate function of macrophages [62]. These data emphasize the critical involvement of ER stress and UPR pathway in terminal effector phases of immune response as well.

### ER Stress and UPR in Type 2 Allergic Response

It has been demonstrated that ER stress and UPR pathways are critically implicated in human allergic diseases and considerable data were derived from studies on allergic inflammation in the lung. In particular, increased levels of ER stress markers (e.g., GRP78, CHOP) were observed in peripheral blood mononuclear cells as well as bronchoalveolar lavage fluids from patients with bronchial asthma compared to those in healthy subjects [63••]. Furthermore, airway epithelial activation of UPR and the related increase of ER-localized chaperones were observed using in vitro HDM-exposed primary human nasal epithelial cells and bronchial epithelial cells [64] and human lung biopsy specimens from asthmatic patients [65]. Interestingly, alleviation of ER stress using chemical chaperones which promote proper folding of client proteins [63, 66] or ablation of UPR and related pathways [64, 65] significantly attenuates allergen-induced typical eosinophilic type 2 immune responses in animal models, thereby highlighting the role of ER stress in inducing and maintaining cardinal features of type 2 immune response.

ER stress and UPR activation can be interconnected with type 2 immune responses through various mechanisms. In an immunological context, ER stress and UPR may lead to transcriptional changes in various cell types essential for the production of certain cytokines/chemokines, thus modulating subsequent immune cell behaviors and inflammatory profiles of tissues [14••]. Moreover, increases of ER-localized chaperones that accompany ER stress is associated with transcriptional activation of UPR that may further potentiate the action of pro-inflammatory, apoptotic, and fibrotic mediators apart from immunologic mechanisms [65]. Importantly, in addition to these classically well-known inflammatory mechanisms associated with ER stress and UPR as substantially reviewed elsewhere [67], a recent study from our group highlights the connection between ER and cellular inflammatory platforms including mitochondria and NLRP3 inflammasome in a unique form of CS-resistant type 2 immune response associated with fungal sensitization [68••].

Respiratory fungal exposure has been regarded as a precipitating factor for severe asthma. A number of epidemiologic studies have shown that fungal sensitization is found more frequently in asthmatics severe asthma [69•, 70, 71]. In one study, over 50% of patients with severe asthma were sensitized to one or more fungi [72]. Fungal sensitization in asthma is often characterized by marked type 2 immune response associated with blood and tissue eosinophilia [2•] and may be associated with airway destruction in the later course of the disease as seen in allergic bronchopulmonary aspergillosis (ABPA) [71]. Based on this knowledge, many researchers have focused on identifying mechanisms whereby fungi can be associated with severe asthma. In our study, GRP78 is remarkably increased in lung tissues from ABPA patients compared to that in healthy subjects. In line with this result, respiratory exposure of mice to fungal allergens from *Aspergillus fumigatus* leads to significant increases in ER stress markers (GRP78 and CHOP) and UPR pathway proteins (phosphorylated (p) IRE1α-XBP1 and p-eIF2α-ATF4). These observations were further verified by in vitro experiments using *A*. *fumigatus*-exposed primary cultured murine tracheal epithelial cells. Notably, *A*. *fumigatus*-induced pulmonary type 2 immune responses, including eosinophilic airway inflammation and increases in the levels of serum total/*A*. *fumigatus*-specific IgE and pulmonary type 2 cytokines (e.g., IL-4, IL-5, and IL-13), are remarkably improved by treatment with a potent ER stress inhibitor. In contrast, dexamethasone fails to improve *A*. *fumigatus*-induced pulmonary type 2 immune responses, implying that ER stress and UPR activation may be implicated in fungus-induced CS-resistant allergic inflammation. Furthermore, our results also demonstrate that oxidative stress, particularly from mitochondria (mitochondrial reactive oxygen species; mtROS) plays a key role in fungal type 2 immune response, and that a potent mtROS scavenger dramatically ameliorates *A*. *fumigatus*-induced CS-resistant type 2 response as well as ER stress [68]. In our unpublished data, respiratory exposure to *A*. *fumigatus* allergens also results in the activation of a cytoplasmic pattern recognition receptor, NLPR3, and subsequent formation of proteolytic multiprotein complex termed inflammasome, an IL-1β producing platform, especially in airway epithelium. Importantly, treatment with anti-IL-1β antibody and/or blockade of activation/assembly of NLRP3 inflammasome using NLRP3-specific inhibitor remarkably ameliorates fungi-induced CS-resistant type 2 immune responses. Considering that ER stress can cause the release of various damage-associated molecular patterns from mitochondria (e.g., mtROS, mitochondrial DNA, ATP, and calcium), which are also potent activators of cytosolic NLRP3 inflammasome [73], interconnection between ER stress, mitochondria, and the NLRP3 inflammasome may play a pivotal role in the pathogenesis of CS-resistant severe type 2 immune response (Fig. 1). In addition, nuclear translocation of NF-κB p65 is remarkably increased in lung tissues from the murine model of *A*. *fumigatus*-induced fungal allergic lung inflammation and an ER stress regulator reduces the *A*. *fumigatus*-induced increase of NF-κB p65 nuclear translocation [68••]. Moreover, treatment of mice with an NF-κB inhibitor reduces the *A*. *fumigatus*-induced type 2 cytokine production and eosinophilic allergic inflammation [68••]. These findings suggest the crucial implication of ER stress-associated NF-κB signaling in fungi-induced CS-resistant type 2 inflammation.

### ER Stress and UPR in Non-type 2 Allergic Response

In lipopolysaccharide (LPS)-induced acute lung inflammatory animal model, ER stress has been reported to be linked to several transcriptional factors including NF-κB and hypoxia-inducible factor 1α, all of which play a central role in acute neutrophil-dominant inflammation and plasma exudation in the lung [41•]. In addition, LPS-induced ER stress leads to the increased expression of IL-17 in airway epithelium, thereby further potentiating ER stress and NF-κB activation via forming a positive feedback loop in airway epithelial cells [74•]. These data suggest that ER stress and UPR pathways may play a role in non-type 2 neutrophilic allergic response. However, the contribution of ER stress and UPR in non-type 2 immune response has been less defined compared to that in type 2 response. Notably, we previously showed that ER stress is critically implicated in the pathogenesis of bronchial asthma, particularly non-type 2 form of the disease, by using ovalbumin (OVA)/LPS-sensitized and OVA/LPS-challenged (OVA_LPS_-OVA) mice [63••]. The classical OVA-sensitized/challenged (OVA-OVA) mice show CS-responsive pulmonary eosinophilic type 2 inflammation. In contrast, the OVA_LPS_-OVA mice display neutrophilic airway inflammation with mixed type 17/type 1/type 2 profiles (i.e., concurrent increases of IL-17/IFN-γ and type 2 cytokines such as IL-4, IL-5, and IL-13 in the lung), all of which are not improved by systemic CS. Interestingly, a potent ER stress regulator significantly reduces the OVA/LPS-induced CS-resistant neutrophilic inflammation as well as ER stress. Meanwhile, increased levels of ER stress and UPR protein are observed in lung tissues of OVA-OVA mice and administration of systemic CS markedly reduces the OVA-induced elevations of these proteins. These data imply the crucial involvement of ER stress and UPR in non-type 2 CS-resistant allergic immune responses. Further studies are needed to delineate their roles in non-type 2 immune responses.

## Conclusions and Perspectives

**Fig. 2:**
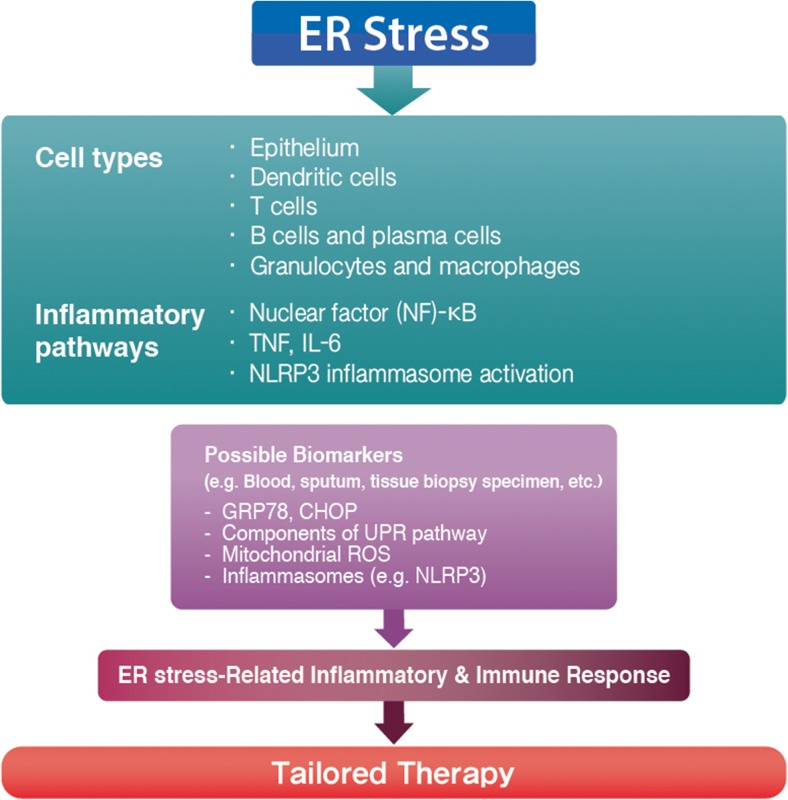
A proposed endotype-driven approach based on endoplasmic reticulum (ER) stress in allergic diseases. ER stress and unfolded protein response (UPR) pathways are closely associated with allergic immune responses involving various important cell types (e.g., epithelial cells, dendritic cells, T and B cells, granulocytes, and macrophages) and inflammatory pathways (e.g., ER stress-associated nuclear factor (NF)-κB signaling, UPR-dependent secretion of interleukin (IL)-6 and tumor necrosis factor (TNF), and NLRP3 inflammasome-mediated IL-1β production). Through analyzing ER stress-associated molecular profiles including ER stress markers (e.g., GRP78 and CHOP), UPR pathway components, and related inflammatory platforms (e.g., mitochondrial reactive oxygen species (ROS) and NLRP3 inflammasome) in blood, sputum, or tissue biopsy specimen from allergic patients, we may design a novel endotype-based approach in association with ER stress

As the beginning of a new era, precision medicine will offer a chance to effectively control and even cure allergic disease for a specific group of patients. Particularly for allergic diseases, current clinical/experimental approaches are largely based on the classification as type 2 and non-type 2 immune responses, and this seems to be quite useful, at least for type 2 response. However, investigation of mechanisms and endotype-driven interventions regarding non-type 2 immune response lags much behind those of type 2 responses. As described earlier, studies have shown that ER stress and UPR pathways may be involved in both types of immune responses via engagement with various important cell types and inflammatory pathways. By analyzing ER stress-associated molecular profiles including ER stress markers (e.g., GRP78 and CHOP), UPR components, and inflammatory platforms (e.g., mitochondrial ROS and NLRP3 inflammasome) in blood, sputum, or tissue biopsy specimen from allergic patients, we may verify the presence and intensity of ER stress and design a novel therapeutic strategy targeting ER stress and specific UPR pathways. We can also monitor the treatment response in these patients through selected biomarkers from ER stress-related pathways. This concept parallels a novel endotype-based approach in allergic disease in association with ER stress (Fig. 2). As CS-resistant allergic inflammation accounts for a significant proportion of the healthcare expenditure for allergic disease, understanding mechanisms behind the close involvement of ER stress in improved knowledge on this issue will permit a great chance to cure intractable allergic disease. Large-scale clinical trials and large cohort studies may be warranted to further delineate the role of ER stress in many human allergic diseases in the future.
